# A series of new *E. coli*–*Thermococcus* shuttle vectors compatible with previously existing vectors

**DOI:** 10.1007/s00792-018-1019-6

**Published:** 2018-03-01

**Authors:** Ryan Catchpole, Aurore Gorlas, Jacques Oberto, Patrick Forterre

**Affiliations:** 1 Unité de Biologie Moléculaire du Gène chez les Extrêmophiles (BMGE), Département de Microbiologie, Institut Pasteur, 25 Rue du Docteur Roux, 75015 Paris, France; 2 Institute for Integrative Biology of the Cell (I2BC), Microbiology Department, CEA, CNRS, Univ. Paris-Sud, Université Paris-Saclay, Gif-sur-Yvette, France

**Keywords:** Archaea, Hyperthermophiles, Gene cloning and expression, Cloning, Molecular biology, Genetics of extremophiles, Molecular biology of archaea

## Abstract

Hyperthermophilic microorganisms are an important asset in the toolkits of biotechnologists, biochemists and evolutionary biologists. The anaerobic archaeon, *Thermococcus kodakarensis,* has become one of the most useful hyperthermophilic model species, not least due to its natural competence and genetic tractability. Despite this, the range of genetic tools available for *T. kodakarensis* remains limited. Using sequencing and phylogenetic analyses, we determined that the rolling-circle replication origin of the cryptic mini-plasmid pTP2 from *T. prieurii* is suitable for plasmid replication in *T. kodakarensis*. Based on this replication origin, we present a novel series of replicative *E. coli*–*T. kodakarensis* shuttle vectors. These shuttle vectors have been constructed with three different selectable markers, allowing selection in a range of *T. kodakarensis* backgrounds. Moreover, these pTP2-derived plasmids are compatible with the single-existing *E. coli*–*T. kodakarensis* shuttle vector, pLC70. We show that both pTP2-derived and pLC70-derived plasmids replicate faithfully while cohabitating in *T. kodakarensis* cells. These plasmids open the door for new areas of research in plasmid segregation, DNA replication and gene expression.

**Electronic supplementary material:**

The online version of this article (10.1007/s00792-018-1019-6) contains supplementary material, which is available to authorized users.

## Introduction

Investigating and understanding the ability of cells to survive in hyperthermophilic environments is an increasingly important undertaking in the fields of biochemistry, biotechnology, molecular biology and evolution, to name just a few. Biochemists have known the value of thermostable enzymes for many decades, particularly in structural studies, where proteins from thermophiles allow unmatched insight into mechanisms of protein stability (Razvi and Scholtz 2006; Pucci and Rooman 2017). Additionally, enzymes derived from extremophiles are often functional in conditions where mesophilic enzymes would quickly degrade, giving them enormous potential in biotechnology (Bruins et al. 2001; Haki and Rakshit 2003; Elleuche et al. 2015). In molecular biology, the unusual biology of archaea and their unique genetic systems have opened our eyes to new and intriguing mechanisms of gene regulation, gene transfer, and metabolic processes (Olsen and Woese 1997; Sato and Atomi 2011; Wagner et al. 2017). Furthermore, the importance of thermophiles in evolutionary biology cannot be understated; thermophiles or hyperthermophiles may be at the base of both the bacterial and the archaeal/eukaryal branches of the tree of life (Boussau et al. 2008; Groussin and Gouy 2011). As a result, understanding the genetics of such hyperthermophiles could have far-reaching implications and applications. Unfortunately, the range of genetic tools and techniques available is very limited. For example, in the extensively studied Thermococcales (anaerobic hyperthermophilic archaea), only a single cloning vector is published (Santangelo et al. 2008), despite over 175 cultivatable strains having been isolated (Lepage et al. 2004; Price et al. 2015) and 26 complete genomes deposited in the Genbank database. As a result, despite *Thermococcus kodakarensis* having been adopted as a model organism, only a single vector is available for the transformation of, and expression of exogenous genes in this species. To study the mechanisms of plasmid maintenance, and to understand the mechanisms of horizontal gene transfer observed in these extremophilic archaea, it is important to have genetic tools which allow us to follow multiple genes, and multiple replicons simultaneously.

Hence, we sought to generate a new *Escherichia coli*–*T. kodakarensis* shuttle vector which is compatible with the only currently available vector, pLC70. In recent years, our group has sequenced 43 plasmids from Thermococcales species (unpublished data), 29 of which co-exist in the same cells as other plasmids or circular viral genomes, proving their compatibility (if plasmid incompatibility exists in Thermococcales). This provided a wide selection of potential origins of replication for use in *T. kodakarensis.*

We present a series of novel *E. coli*–*T. kodakarensis* shuttle vectors based on the small cryptic plasmid pTP2 from *T. prieurii,* and the *E. coli* p15A origin of replication. This plasmid backbone has been developed in combination with three different markers for selection in *T. kodakarensis* strains. Additionally, we show that this plasmid is compatible with the single published cloning vector for Thermococcales, pLC70 (and derivatives thereof).

## Materials and methods

### Strains and media

Plasmid construction was carried out in *Escherichia coli* strain XL1-Blue grown at 37 °C in LB medium. Where necessary, media was supplemented with Ampicillin (100 µg/mL), Kanamycin (40 µg/mL) or Chloramphenicol (20 µg/mL). All archaeal work was carried out in *Thermococcus kodakarensis* strain TS559 (Santangelo et al. 2010) grown at 85 °C in either ASW-YT (Sato et al. 2003) or ASW-CH medium with uracil supplementation (10 µg/mL) (Fujikane et al. 2010). Where necessary, media was supplemented with agmatine sulfate (1.0 mM) or mevinolin (10 μM).

### Plasmid construction

For a complete list of strains and plasmids used in this study, see Supplementary Table 1.

Plasmid pTPTK1 was constructed by Gibson Assembly using the NEBuilder HiFi DNA Assembly Master Mix (New England Biolabs) following the manufacturer’s protocol. Briefly, the *E. coli* p15A origin of replication was amplified by PCR from the plasmid pBAD33 (kindly gifted by Alicia Lai, University of Canterbury) using primers ‘pTPTK1.GA.1’ and ‘pTPTK1.GA.2’ (for primer sequences, see Supplementary Table 2). The HMG-CoA cassette (conferring resistance to mevinolin in *T.* *kodakarensis*) was amplified by PCR from the plasmid pLC70 (kindly gifted by Thomas Santangelo, Colorado State University) using primers ‘pTPTK1.GA.5’ and ‘pTPTK1.GA.6’. The entire sequence of pTP2 was amplified by PCR from *Thermococcus prieurii* DNA using the primers ‘pTPTK1.GA.3’ and ‘pTPTK1.GA.4’. PCR products were purified, assembled, and used to transform *E. coli* strain XL1-Blue. Transformants were selected by growth in the presence of chloramphenicol and confirmed by Sanger sequencing (Beckman Genomics).

Plasmids pTPTK2 and pTPTK3 were constructed using pTPTK1 as a starting point. Briefly, the pBAD33-pTP2 backbone of pTPTK1 was amplified by PCR using primers ‘pTPTK2/3.GA.1’ and ‘pTPTK2/3.GA.2’. For pTPTK2, the *trpE*-cassette (conferring tryptophan prototrophy to *T. kodakarensis ΔTK0254* backgrounds) was amplified from the plasmid pLC70 using primers ‘pTPTK2.GA.3’ and ‘pTPTK2.GA.4’. For pTPTK3, the gene *TK0149* (conferring agmatine prototrophy to *T. kodakarensis ΔTK0149* backgrounds) was amplified from the chromosome of *T. kodakarensis* KOD1 along with its native promoter using primers ‘pTPTK3.GA.3’ and ‘pTPTK3.GA.4’. PCR products were assembled and sequenced as above.

Plasmid pTNAg was constructed by assembling the *TK0149* cassette (PCR-amplified from the *T.* *kodakarensis* KOD1 chromosome using primers ‘pTNAg.GA.1’ and ‘pTNAg.GA2’) with the *Apa*I-*EcoR*V digestion product of pLC70. The resulting plasmid was selected in *E. coli* by growth in the presence of ampicillin and kanamycin and confirmed by Sanger sequencing (Beckman Genomics).

Plasmid pTNTrpE was constructed by blunting and circularization of the *Xba*I–*EcoR*V fragment of pLC70. The absence of the HMG-CoA cassette was confirmed by Sanger sequencing (Beckman Genomics).

### Transformation of *T. kodakarensis*

Transformation was carried out as described previously (Sato et al. 2005). Briefly, ~ 5 × 10^8^ late exponential phase *T. kodakarensis* TS559 cells were harvested under anaerobic conditions by centrifugation at 4000×*g* for 10 min. The cell pellet was resuspended in 200 µL 0.8×ASW, and 5 µg of plasmid DNA was added. Suspensions were incubated on ice for 60 min, heat shocked at 85 °C for 60 s, then chilled on ice for 10 min. 1 mL ASW-YT + agmatine was added, and the cultures were incubated at 85 °C for 1.5 h. Cells were harvested by centrifugation at 4000×*g* for 3 min and used to inoculate 25 mL of selective media. Transformant cultures were grown at 85 °C for 48 h before being sub-cultured twice by 1:100 dilution in fresh selective media. Transformation was confirmed by isolation of plasmid DNA from 20 to 50 mL of culture (plasmid DNA recovered using Macherey–Nagel NucleoSpin Plasmid kit) and analysis by restriction digestion and gel electrophoresis.

Double transformation of *T. kodakarensis* TS559 was performed sequentially, i.e. plasmid-containing cultures were grown in selective media, and transformed with a second plasmid. Following selection and sub-culturing of double transformants, serial dilutions of stationary phase cultures were suspended in a solution of molten 0.7% Gelrite containing 10 g/L colloidal sulfur and plated as a top layer on solid selective media (under anaerobic conditions). Single colonies (observable by local clearing of the colloidal sulfur) were used to inoculate selective liquid media.

### Liposome-mediated transformation

Transformation was performed using a modification of a method previously described (Metcalf et al. 1997). Briefly, cultures of Thermococcales species were grown to late-log phase in ASW-YT medium. Cells were pelleted at 4000×*g* for 10 min under anaerobic conditions, and resuspended in 0.1 vol of 0.85 M sorbitol (de-gassed and reduced with Na_2_S). For each transformation, liposomes were generated by adding 15 μL DOTAP to 150 μL 20 mM HEPES, pH 7.4, with 2 µg DNA. The liposome suspension was added to 1 mL resuspended culture, and incubated at room temperature for 1 h. Cells were pelleted and resuspended in 1 mL ASW-YT medium, then allowed to recover for 2 h at 85 °C. Cells were again pelleted and used to inoculate 25 mL selective medium.

### Phylogenetic analysis

Sequences for replication-associated proteins of pTN1 (rep74, ABR10429.1) and pTP2 (repTP2, YP_007974244.1) were used as query sequences for a BLAST (Altschul et al. 1990), Phyre2 (Kelley et al. 2015) or HHpred (Söding et al. 2005) search. The top BLAST hits were aligned using MSAprobs v0.9.7 (Liu and Schmidt 2014), gaps removed using BMGE v1.12 (Criscuolo and Gribaldo 2010), amino acid substitution models determined by ModelFinder (Kalyaanamoorthy et al. 2017), and phylogenetic trees generated with IQ-TREE v1.5.4 (Nguyen et al. 2015). Trees were visualized with iTOL v3.6.1 (Letunic and Bork 2016).

## Results

### Identification of a suitable origin of replication

As pLC70 encodes the origin of replication from plasmid pTN1 of *Thermococcus* *nautili* (Soler et al. 2007; Santangelo et al. 2008), the most obvious place to search for a plasmid with a compatible origin of replication is the other co-existing plasmids in *T. nautili*. However, with pTN2 and pTN3 comprising 13 and 18 kb, respectively, they are less than ideal candidates for the design of small, easily manipulated shuttle vectors.

**Fig. 1:**
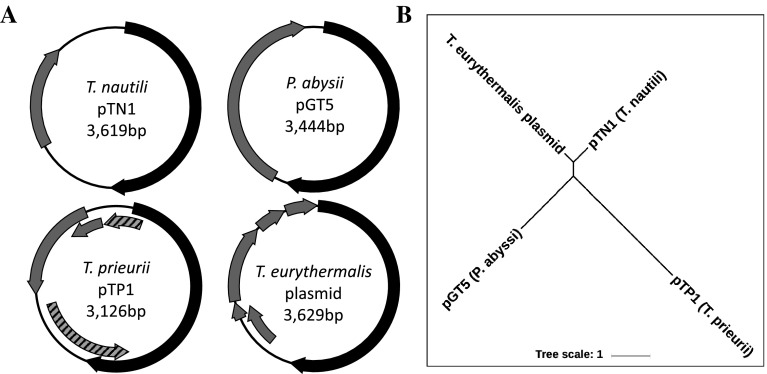
**a** Schematic diagrams of pTN1-family plasmids. Predicted ORFs are indicated by gray blocks. ORFs encoding predicted replication-associated proteins are black. In the case of *T. prieurii*, the complete Rep gene interrupts another Rep-like gene colored in gray with black stripes. **b** Phylogenetic relationship between pTN1-family Rep proteins. Unrooted phylogenetic tree generated with the full-length Rep proteins of the 4 pTN1-family plasmids

Phylogenetic analysis of the replication-associated protein encoded by pTN1 reveals it to be part of a small family of homologous genes encoded by other Thermococcales plasmids (Fig. 1). One of the rep genes identified in this analysis is that of the small cryptic plasmid pTP1 from *T. prieurii* (Gorlas et al. 2013a, b). The rep protein of plasmid pTP1 (RepTP1, YP_007974239) is 31.3% identical to that of pTN1 (Rep74, ABR10429) at the amino acid level. This similarity, combined with the close phylogenetic relatedness of these two species (Gorlas et al. 2014) suggested that the replication-associated proteins are likely homologs belonging to the same family of archaeal plasmids. It is notable that pTP1 is unusual in structure, likely having acquired its replication protein via recombination with another mobile element (Gorlas et al. 2013b). Thus, while the other ORFs of pTP1 differ from the usual organization of pTN1-family plasmids, the Rep protein is clearly homologous to that of pTN1. We, therefore, predicted that co-existing plasmids from *T. prieurii* may encode replication origins that could be compatible with pTN1 (and, therefore, pLC70). *T. prieurii* contains three circular extra-chromosomal elements—the genome of the virus TPV1 (21,592 bp), and two small cryptic plasmids, pTP1 and pTP2 (3126 and 2038 bp, respectively) (Gorlas et al. 2013b). Due to its small size and compatibility with pTP1, pTP2 was chosen as a potentially suitable origin of replication for shuttle vector construction.

### Analysis of pTP2-rep

While pTP2 is the smallest known Thermococcales plasmid, it appears to have a surprisingly complicated gene structure. Whereas the pTN1-family of Thermococcales plasmids are quite simple in structure (usually containing only two ORFs, one of which encodes the replication-associated protein), pTP2 encodes several putative transcriptional regulators, as well as a transmembrane domain protein (Gorlas et al. 2013b). The function of these proteins in the maintenance and potential transmission of pTP2-family plasmids, or indeed secondary functions, remains unknown. The predicted replication-associated protein of pTP2 is also enigmatic. This protein shares 30–35% sequence identity with a protein of unknown function found in the genomes of multiple species of methanogenic Euryarchaeota. Both structural and functional prediction (using Phyre2 and HHpred, respectively) of these methanogen genes, and of RepTP2 itself reveals the central ~ 120 residues of these proteins to be similar to replication-associated proteins of various mobile elements infecting all three domains of life, e.g. SIRV1 from the archaeon *Sulfolobus islandicus*; pMV158 from the bacterium *Streptococcus agalactiae*; and TYLSCV from the eukaryote *Solanum lycopersicum* (Supplementary Table 3). The similarity of both RepTP2 and other mobile elements to chromosomally-encoded genes of methanogenic Euryarchaeota may suggest that these genes are part of integrated horizontally mobile elements in these species. We were unable to find any synteny of the genes surrounding these Rep-like proteins, indicating that these proteins may have arisen from multiple different integrated elements, or that the similarity in sequence, structure and predicted function is due to a similarity of function, e.g. a native methanogen protein encoding a replication-associated functionality (helicase, polymerase, resolvase, etc).

### Generation of shuttle vectors

**Fig. 2:**
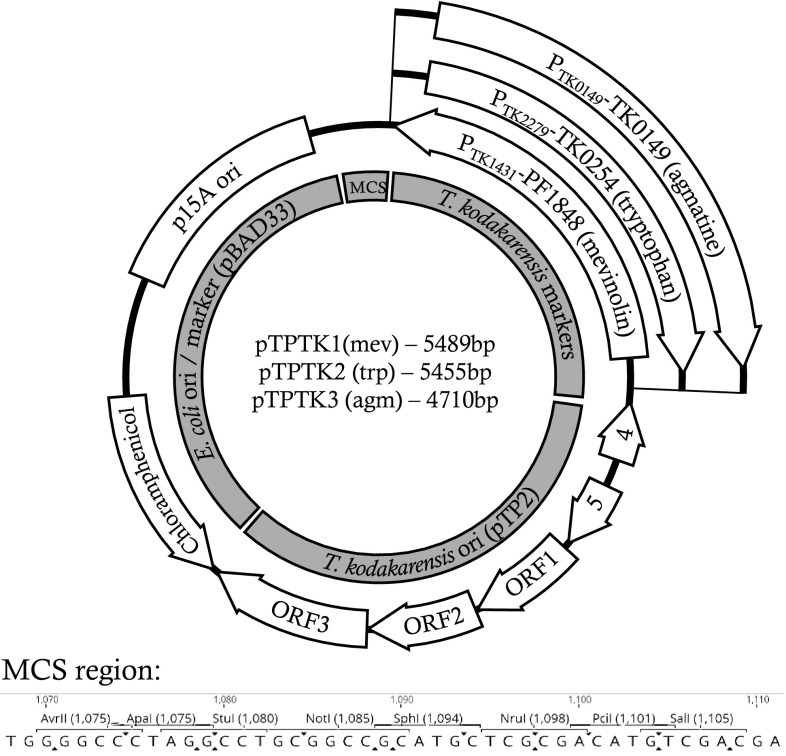
Plasmid maps of pTPTK1, pTPTK2 and pTPTK3. ORFs/genetic elements are indicated by white boxes outlined in black with labels indicating the nature of each element. Gray regions inside the plasmid map indicate the source and nature of each plasmid region

Plasmid pTP2 encodes five predicted ORFs, three of which are overlapping. Therefore, the possible sites where the plasmid could be linearized without disrupting potential gene activity are limited. The intergenic space between ORF3 and ORF4 (Fig. 2) was chosen as it contains a 27-bp non-coding region, as well as being at the 3′ termini of both ORF3 and ORF4, thus decreasing the chance of disrupting upstream regulatory sequences such as promoters. Intriguingly, linearization of pTP2 in this region resulted in a ~ 1.8-Kbp fragment, as determined by gel electrophoresis (data not shown), rather than the 2038-bp expected from the published sequencing data. Sequencing of the fragment revealed that the linearized pTP2 was missing a tandem repeat corresponding to nucleotides 710–907 and 913–1110 of sequence NC_021208.1 (Supplementary Figure 1). It has not been confirmed whether this fragment was lost in the pTP2 isolate used as a template, or during PCR.

To minimize the risk of recombination between a pTP2-derived vector and pLC70 while maintained within a single cell, an *E. coli* vector backbone and antibiotic-resistant marker were chosen which are different to those used in generating pLC70. Where pLC70 is comprised of a pUC origin of replication in combination with ampicillin- and kanamycin-resistant genes (derived from pCR2.1-TOPO), a p15A origin of replication was chosen for our shuttle vector, in combination with a chloramphenicol-resistant gene (both derived from pBAD33).

The range of selectable markers available for *T. kodakarensis* is very limited, comprising genes which complement strain-specific auxotrophies, e.g. tryptophan or agmatine; or a single antibiotic resistance determinant produced by HMG-CoA reductase overexpression providing resistance to mevinolin. To maximize the usefulness of an *E. coli*–*T. kodakarensis* shuttle vector, we generated three different variants of the pTP2-derived vector, each encoding a different selectable marker.

All three vectors contain an identical backbone as described above, comprising the 1.8-kb pTP2 sequence, p15A origin, and chloramphenicol-resistant marker (Fig. 2). The following marker cassettes were added for selection in *T. kodakarensis:* pTPTK1 encodes HMG-CoA reductase under the constitutive glutamate dehydrogenase promoter (sourced from pLC70 (Santangelo et al. 2008)), conferring resistance to mevinolin; pTPTK2 encodes anthranilate synthase (TK0254 ≡ *trpE*) under the control of the promoter from CDP-alcohol phosphatidyltransferase (TK2279) (also sourced from pLC70), conferring tryptophan prototrophy to *trpE* mutant backgrounds; pTPTK3 encodes pyruvoyl-dependent arginine decarboxylase (TK0149) under its native promoter, conferring agmatine prototrophy to TK0149 mutant backgrounds.

A small multiple cloning site (MCS) site was added to the shuttle vector design (Fig. 2) to aid in the cloning of additional markers and/or genes. A limited number of enzyme recognition sequences were available which neither cut the vector backbone, nor the potential selectable markers; therefore, the MCS comprises 40 bp with sites for *Apa*I, *Avr*II, *Stu*I*, *Not*I, *Sph*I, *Nru*I, *Pci*I and *Sal*I* (*StuI is not suitable for cloning in pTPTK2 as there is a site within *TK0254.* Additionally, although still suitable for cloning, a second *Sal*I site is present at the junction between the vector and TK0254 cassette, resulting in *Sal*I digestion releasing a 24-bp fragment from the vector).

### Replication in *T. kodakarensis*

**Fig. 3:**
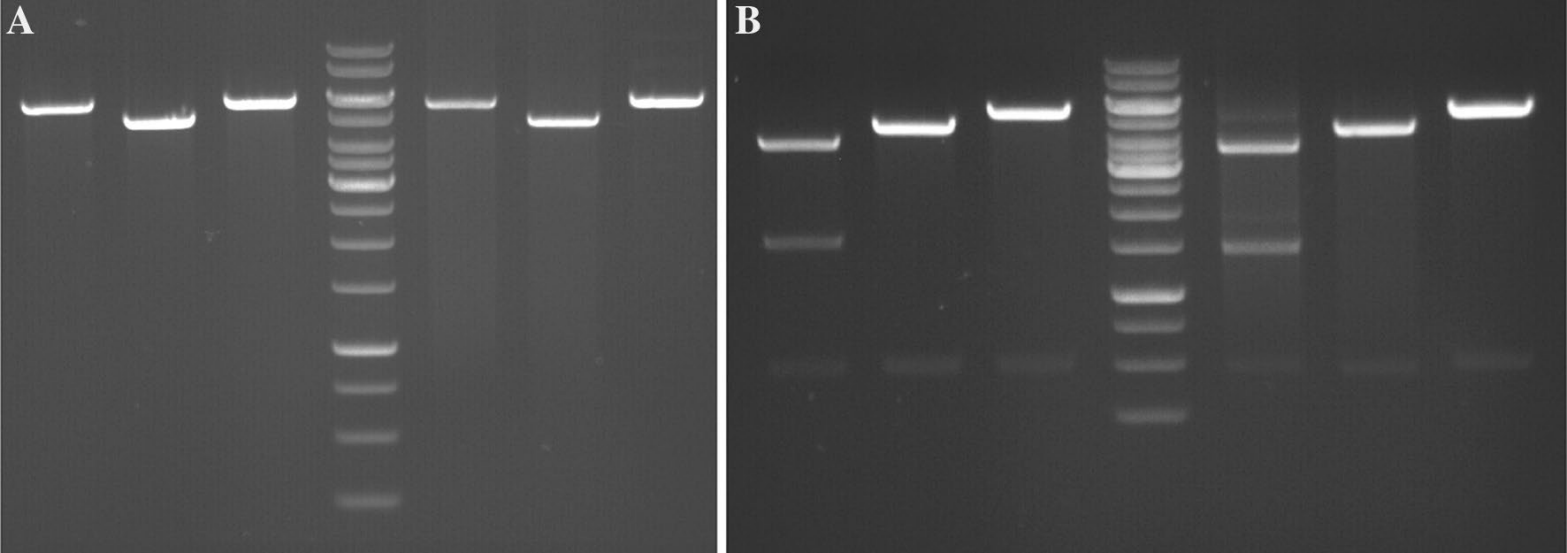
Digestion and gel electrophoresis of pTP2-derived plasmids. **a** Plasmids digested with *Rru*I, recognizing a single site on each plasmid. Lane 1: pTPTK1 isolated from *E. coli*, lane 2: pTPTK3 isolated from *E. coli,* lane 3: pTPTK2 isolated from *E. coli,* lane 4: GeneRuler 1 kb DNA ladder, lane 5: pTPTK1 isolated from *T. kodakarensis,* lane 6: pTPTK3 isolated from *T. kodakarensis,* lane 7: pTPTK2 isolated from *T. kodakarensis.* **b** Plasmids digested with *Hind*III, recognizing multiple sites on each plasmid. Lane 1: pTPTK1 isolated from *E. coli,* lane 2: pTPTK3 isolated from *E. coli,* lane 3: pTPTK2 isolated from *E. coli,* lane 4: GeneRuler 1 kb DNA ladder, lane 5: pTPTK1 isolated from *T. kodakarensis,* lane 6: pTPTK3 isolated from *T. kodakarensis,* lane 7: pTPTK2 isolated from *T. kodakarensis*

Following construction of the three plasmids in *E. coli*, they were used to transform *T. kodakarensis* strain TS559 (Santangelo et al. 2010). Initial transformations were performed in liquid culture, and each of the three plasmids conferred the appropriate prototrophy/resistance (mevinolin resistance for pTPTK1; tryptophan prototrophy for pTPTK2; agmatine prototrophy for pTPTK3). Transformant cultures were able to form discreet colonies on solid selective media. Plasmid DNA isolated from transformant cultures (both from original transformations following two sub-cultures, and from cultures inoculated with single colonies) gave a restriction digestion pattern identical to that observed with plasmids isolated from *E. coli* suggesting these plasmids were faithfully replicated in *T. kodakarensis* (Fig. 3). Additionally, re-transformation of *E. coli* using plasmid DNA isolated from *T. kodakarensis* transformant cultures successfully re-introduced the plasmid and chloramphenicol marker.

### Compatibility with pLC70-derived plasmids

To assess the compatibility of pTP2-derived plasmids with pLC70-derived plasmids, it was necessary to select for both plasmids in a single culture. However, with pLC70 encoding both tryptophan prototrophy and mevinolin-resistant markers, it was necessary to modify pLC70 to have different selectable markers on the co-transforming plasmids. Plasmid pTNAg was generated by deletion of the entire *TK0254*-*PF1848* (*TrpE *− HMG-CoA) cassette from pLC70, and replacement with the *TK0149* gene encoding pyruvoyl-dependent arginine decarboxylase (for which *T. kodakarensis* TS599 is a knockout). Plasmid pTNTrpE was generated by deletion of *PF1848* from pLC70, resulting in a plasmid encoding the *TrpE* marker alone. Both of these plasmids were able to replicate faithfully in *T. kodakarensis* (Supplementary Figure 2), conferring agmatine and tryptophan prototrophy, respectively, to strain TS559.

Double transformants of *T. kodakarensis* were generated by transforming a strain harboring a pLC70-derived plasmid with a pTP2-derived plasmid, or vice versa. Although the transformation rate was not quantified, transformation was successful regardless of the nature of the incumbent or incoming plasmid. However, for technical reasons, it is most simple to carry out the double transformation such that the incumbent plasmid is one which can be selected in rich media, i.e. conferring agmatine prototrophy (pTPTK3 or pTNAg) or mevinolin resistance (pTPTK1 or pLC70), thus ensuring a cell-dense, exponential phase culture can be readily produced for the second transformation.

**Fig. 4:**
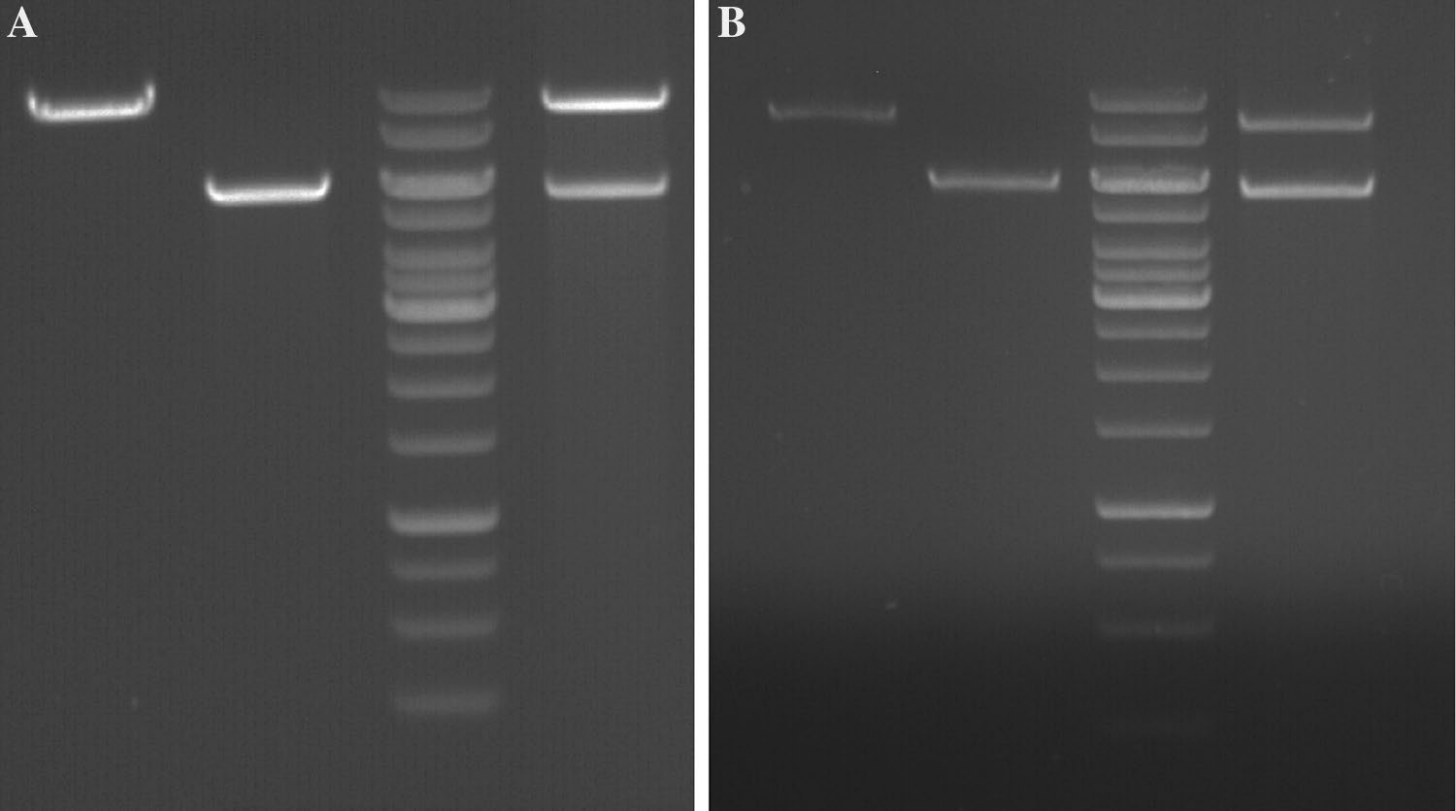
Digestion and gel electrophoresis of plasmids from double transformants. **a** Plasmids digested with *Rru*I, recognizing a single site on each plasmid. Lane 1: pTNTrpE isolated from *E. coli,* lane 2: pTPTK1 isolated from *E. coli,* lane 3: GeneRuler 1 kb DNA ladder, lane 4: pTNTrpE + pTPTK1 isolated from *T. kodakarensis* double transformant. **b** Plasmids digested with *Rru*I, recognizing a single site on each plasmid. Lane 1: pTNAg isolated from *E. coli,* lane 2: pTPTK2 isolated from *E. coli,* lane 3: GeneRuler 1 kb DNA ladder, lane 4: pTNAg + pTPTK2 isolated from *T. kodakarensis* double transformant

Transformation with two plasmids conferred upon cultures the appropriate prototrophies and/or resistance. DNA extracted from double-transformant cultures indeed gave two plasmid bands upon electrophoresis, and plasmids produced the appropriate digestion patterns (Fig. 4). Plasmid extractions were able to transform *E. coli* to chloramphenicol resistance and ampicillin/kanamycin resistance. Furthermore, dilutions of plasmid extractions resulted in transformation of *E. coli* to chloramphenicol resistance while maintaining ampicillin sensitivity, and vice versa, indicating that the two plasmids were replicating faithfully and separately in the *T. kodakarensis* host. To confirm that *T. kodakarensis* cultures were indeed double transformants, rather than a mixed culture of single transformants, cultures were plated to single colonies on solid selective medium. Following incubation, single colonies were suspended in 0.8 × ASW, vortexed vigorously, and serial dilutions plated again on solid selective medium. Recovered colonies grew well in selective medium, and all gave two plasmids upon DNA extraction and electrophoresis or *E. coli* transformation.

It should be noted that selection for both tryptophan prototrophy and mevinolin resistance (ASW-CH + mevinolin) resulted in slow-growing cultures, likely due to an increased sensitivity of cells to mevinolin in the less-rich ASW-CH medium over the rich ASW-YT medium.

### Host-range of pTP2-derived vectors

To assess the ability of pTP2-derived vectors to replicate in other Thermococcales species, we attempted to use pTPTK1 to transform *T. aggregans, T. pacificus, T. siculi, T. celer, T. guaymasensis, T. fumicolans* and *T. prieurii.* As no method of transformation has been established for these species, we attempted transformation using the method described for *T. kodakarensis.* This method relies on the natural competence of *T. kodakarensis*, and so it is perhaps unsurprising that we were unable to obtain transformants of these untested species (only *T. kodakarensis* gave transformants with this protocol). We then attempted transformation using DNA encapsulated in liposomes, as previously described for other Euryarchaeota such as Methanosarcina (Metcalf et al. 1997) and *Methanococcus voltae* (Sniezko et al. 1998), as well as hyperthermophilic bacteria of the genus Thermotoga (Yu et al. 2001). Unfortunately, this method did not yield transformants in any Thermococcus species tested, even *T. kodakarensis.* Transformation was also attempted of the naturally competent *Pyrococcus furiosus* COM1 strain; however, no plasmid-containing transformants were obtained.

## Discussion

We have established that the cryptic plasmid pTP2 from *Thermococcus prieurii* encodes an origin of replication which is functional in *T. kodakarensis.* We have used this origin of replication to generate a series of *E.* *coli*–*T. kodakarensis* shuttle vectors. These vectors replicate stably (under selection) in both *T. kodakarensis* and *E. coli*. Importantly, these vectors are compatible with the previously described *E. coli*–*T. kodakarensis* shuttle vector, pLC70 (Santangelo et al. 2008), both in *E.* *coli* and in *T.* *kodakarensis,* greatly contributing to the range of genetic tools available for Thermococcales. Further work will be necessary to quantify the relative copy numbers of pTN1-derived and pTP2-derived plasmids when co-maintained in *T.* *kodakarensis;* however, visual analysis of plasmids by gel electrophoresis suggests that they do not differ greatly.

The ability of both pTP2-derived (e.g. pTPTK1) and pTN1-derived plasmids (e.g. pLC70) to replicate concurrently and faithfully inside a single *T. kodakarensis* strain suggests that pTP2 and pTN1 belong to separate families of plasmids within the Thermococcales. Indeed, sequence analysis of their replication-associated proteins shows no obvious similarities in sequence or predicted structure, suggesting that they likely function independently. Furthermore, pTP2 is found naturally with a pTN1-like plasmid, pTP1, proving their ability to be maintained together, even in the absence of selectable markers.

We were unable to successfully introduce a pTP2-derived plasmid (pTPTK1) into any other *Thermococcus* species. It remains unclear whether this failure in transformation is due to a failure of the plasmid DNA to enter the cell, or a failure of the plasmid to replicate once inside the cell. Our inclusion of *T. prieurii* in these experiments suggests that, at least for this species, the failure is a lack of DNA entry, as *T.* *prieurii* is the natural host for pTP2.

The range of cultivable and genetically tractable Archaea for study as model organisms is extremely limited. Similar limitations exist in the study of hyperthermophilic organisms, which are proving to have great biotechnological potential. Thus, *T.* *kodakarensis—*a hyperthermophilic archaeon with an established range of genetic tools*—*represents an important model species. The ability to transform *T. kodakarensis* with two different and stable extra-chromosomal replicons will open up new fields of study in these important organisms, e.g. plasmid partitioning/segregation and the related plasmid compatibility/incompatibility, DNA-binding proteins together with their substrates, alpha-complementation of enzymes, etc.

## Electronic supplementary material

Below is the link to the electronic supplementary material.

Supplementary material 1 (DOCX 15 kb)

Supplementary material 2 (DOCX 61 kb)

Supplementary material 3 (DOCX 116 kb)

Supplementary material 4 (PDF 37 kb)

Supplementary material 5 (PDF 519 kb)
