# Supplementary material for: A series of new E. coli–Thermococcus shuttle vectors compatible with previously existing vectors
Source: Extremophiles. 2018 Mar 1;22(4):591–8. doi: 10.1007/s00792-018-1019-6 (PMC5988781; doi:10.1007/s00792-018-1019-6)
Supplement: Supplementary file 1 — Supplementary material 1 (DOCX 15 kb) [file 792_2018_1019_MOESM1_ESM.docx]

**Supplementary Table 1**: Strains and plasmids used in this work

| Strain name | | Genotype | | Markers | | Source |
| --- | --- | --- | --- | --- | --- | --- |
| *Escherichia coli* XL1-Blue | | *endA*1 *gyrA*96 thi-1 *recA*1 *relA*1 lac *glnV*44 F'[ ::Tn10 *proAB^+^ lacI^q^*Δ(l*acZ*)M15] *hsdR*17 | | Tetracycline resistance Nalidixic acid resistance | | Stratagene |
| *Thermococcus kodakarensis* TS559 | | Δ*pyrF*; Δ*trpE::pyrF*, Δ*TK0664*, Δ*TK0149* | | Uracil prototrophy Tryptophan auxotrophy Agmatine auxotrophy 6-methylpurine resistant | | Santangelo 2010 |
| Plasmid name | Genotype | | *E. coli* marker(s) | | *T. kodakarenesis* marker(s) | Source (Accession No.) |
| pLC70 | see reference | | AmpR, KanR | | Trp, MevR | Santangelo 2008 (N/A) |
| pTP2 | see reference | | - | | - | Gorlas 2013 (KC617921) |
| pBAD33 | see reference | | CmR | | - | Guzman 1995 (N/A) |
| pTPTK1 | pTP2::(p15A*-cat*),(P_TK1431_-*PF1848*) | | CmR | | MevR | *this work* (MG920815) |
| pTPTK2 | pTP2::(p15A*-cat*),(P_TK2279_-*TK0254*) | | CmR | | Trp | *this work* (MG920816) |
| pTPTK3 | pTP2::(p15A*-cat*),(P_TK0149_-*TK0149*) | | CmR | | Agm | *this work* (MG920817) |
| pTNAg | pLC70Δ(*TK0254-PF1848*):: (P_TK0149_-*TK0149*) | | AmpR, KanR | | Agm | *this work* (MG920813) |
| pTNTrpE | pLC70Δ*PF1848* | | AmpR, KanR | | Trp | *this work* (MG920814) |

AmpR=ampicillin resistance; KanR=kanamycin resistance; CmR=chloramphenicol resistance; Trp=tryptophan prototrophy in a Δ*trpE* (TK0254) background; MevR=mevinolin resistance; Agm=agmatine prototrophy in a ΔTK0149 background.
