# Supplementary material for: A series of new E. coli–Thermococcus shuttle vectors compatible with previously existing vectors
Source: Extremophiles. 2018 Mar 1;22(4):591–8. doi: 10.1007/s00792-018-1019-6 (PMC5988781; doi:10.1007/s00792-018-1019-6)
Supplement: Supplementary file 2 — Supplementary material 2 (DOCX 61 kb) [file 792_2018_1019_MOESM2_ESM.docx]

**Supplementary Table 2**: Primers used in this work

| Primer name | Sequence (5ʹ-3ʹ) |
| --- | --- |
| pTPTK1.GA.1 | CGTAGCACCAGGCGTTTAAG |
| pTPTK1.GA.2 | gtcgacatgtcgcgagcatgcggccgcaggcctagggcccCAGACAATTGACGGCTTGAC |
| pTPTK1.GA.3 | ttcatctagaGGGTTTGAGAAGAGAAGAAAAGGGTTCTCAG |
| pTPTK1.GA.4 | cttaaacgcctggtgctacgTCACGTCGGAGGGGGACCCC |
| pTPTK1.GA.5 | gtcaagccgtcaattgtctggggccctaggcctgcggccgcatgctcgcgacatgtcgacGATATCCATATCGGATCCTC |
| pTPTK1.GA.6 | tctcaaacccTCTAGATGAACTTTGATGAACG |
| pTPTK2/3.GA.1 | GGATCCGATATGGATATCGTC |
| pTPTK2/3.GA.2 | GGGTTTGAGAAGAGAAGAAAAG |
| pTPTK2.GA.3 | acgatatccatatcggatccGTCGACTGCCGCAACGCGCA |
| pTPTK2.GA.4 | tttcttctcttctcaaacccTCATTCCCTCACCCCCAGCGC |
| pTPTK3.GA.3 | acgatatccatatcggatccTCGTTCTCTACTCAATGGCATC |
| pTPTK3.GA.4 | tttcttctcttctcaaacccTCAGTAGGGGAACATGACGAC |
| pTNAg.GA.1 | taagggcgaattctgcagatTCGTTCTCTACTCAATGG |
| pTNAg.GA.2 | actatagggcgaattgggccTCAGTAGGGGAACATGAC |

Uppercase indicates identity to the template sequence (see methods section); lowercase indicates primer extension for Gibson assembly; underline indicates synthetically produced MCS.
