# Supplementary material for: A series of new E. coli–Thermococcus shuttle vectors compatible with previously existing vectors
Source: Extremophiles. 2018 Mar 1;22(4):591–8. doi: 10.1007/s00792-018-1019-6 (PMC5988781; doi:10.1007/s00792-018-1019-6)
Supplement: Supplementary file 3 — Supplementary material 3 (DOCX 116 kb) [file 792_2018_1019_MOESM3_ESM.docx]

**Supplementary Table 3**: A selection of similarity search results using BLASTp, Phyre2 or HHpred, with RepTP2 as a query sequence.

| **BLAST Hits** | | | | |
| --- | --- | --- | --- | --- |
| **Accession** | **Species** | **Query coverage** | **Identity** | **E-value** |
| WP_013179516.1 | *Methanococcus voltae* | 88% | 42% | 1x10^-51^ |
| [WP_013179456.1](https://www.ncbi.nlm.nih.gov/protein/502944480?report=genbank&log$=prottop&blast_rank=3&RID=YPCN70B0014) | *Methanococcus voltae* | 88% | 42% | 1x10^-50^ |
| [KQM09910.1](https://www.ncbi.nlm.nih.gov/protein/944557198?report=genbank&log$=prottop&blast_rank=4&RID=YPCN70B0014) | *Methanomassiliicoccales* archaeon RumEn M2 | 82% | 38% | 3x10^-32^ |
| [AKB35514.1](https://www.ncbi.nlm.nih.gov/protein/805355443?report=genbank&log$=prottop&blast_rank=5&RID=YPCN70B0014) | *Methanosarcina siciliae* C2J | 79% | 34% | 5x10^-25^ |
| [AAM05985.1](https://www.ncbi.nlm.nih.gov/protein/19916572?report=genbank&log$=prottop&blast_rank=6&RID=YPCN70B0014) | *Methanosarcina acetivorans* C2A | 84% | 34% | 5x10^-25^ |
| [WP_048180372.1](https://www.ncbi.nlm.nih.gov/protein/851317232?report=genbank&log$=prottop&blast_rank=7&RID=YPCN70B0014) | *Methanosarcina siciliae* | 79% | 34% | 6x10^-25^ |
| [WP_048065454.1](https://www.ncbi.nlm.nih.gov/protein/851004003?report=genbank&log$=prottop&blast_rank=8&RID=YPCN70B0014) | *Methanosarcina acetivorans* | 84% | 34% | 1x10^-24^ |
| [WP_048155308.1](https://www.ncbi.nlm.nih.gov/protein/851291625?report=genbank&log$=prottop&blast_rank=9&RID=YPCN70B0014) | *Methanosarcina sp.* Kolksee | 80% | 32% | 1x10^-23^ |
| [WP_048159110.1](https://www.ncbi.nlm.nih.gov/protein/851295639?report=genbank&log$=prottop&blast_rank=10&RID=YPCN70B0014) | *Methanosarcina sp.* WWM596 | 79% | 33% | 3x10^-23^ |
| [WP_054299167.1](https://www.ncbi.nlm.nih.gov/protein/932221846?report=genbank&log$=prottop&blast_rank=11&RID=YPCN70B0014) | *Methanosarcina flavescens* | 79% | 32% | 2x10^-22^ |
| **Phyre2 Hits** | | | | |
| **PDB ID** | **Protein structure** | **Coverage** | **ID** | **Confidence** |
| 1L5I | DNA binding domain of the replication initiation protein from a Geminivirus (*Tomato Yellow Leaf Curl Virus-Sardinia*) | 46% | 14% | 97.3% |
| 2X3G | Hypothetical protein ORF119 from *Sulfolobus islandicus* rod-shaped virus 1 | 26% | 24% | 94.7% |
| 1M55 | Catalytic domain of the Adeno Associated Virus type 5 Rep protein | 42% | 15% | 73.0% |
| **HHpred Hits** | | | | |
| **PDB ID** | **Protein structure** | **Coverage** | **ID** | **P-Value** |
| 2X3G | Hypothetical protein ORF119 (*Sulfolobus islandicus rod-shaped virus 1*) | 50% | 20% | 7.6x10^-6^ |
| 4R94 | Nickase domain of NS1 from MVM complexed with magnesium | 18% | 20% | 8.6x10^-5^ |
| 1L2M | DNA-binding domain of the replication initiation protein from a geminivirus (*Tomato yellow leaf curl virus-Sardinia*) | 9% | 22% | 5.4x10^-5^ |
| 4ZO0 | AAV-2 Origin Binding Domain (*Adeno-associated dependoparvovirus A*) | 24% | 16% | 4.1x10^-4^ |
| 4PP4 | Minute virus of mice non-structural protein-1N-terminal nuclease domain (*Rodent protoparvovirus 1*) | 18% | 20% | 1.3x10^-4^ |
| 4LVL | MobM Relaxase Domain (*Streptococcus agalactiae*) | 24% | 14% | 1.6x10^-4^ |
