# Supplementary material for: A series of new E. coli–Thermococcus shuttle vectors compatible with previously existing vectors
Source: Extremophiles. 2018 Mar 1;22(4):591–8. doi: 10.1007/s00792-018-1019-6 (PMC5988781; doi:10.1007/s00792-018-1019-6)
Supplement: Supplementary file 5 — Supplementary material 5 (PDF 519 kb) [file 792_2018_1019_MOESM5_ESM.pdf]

A

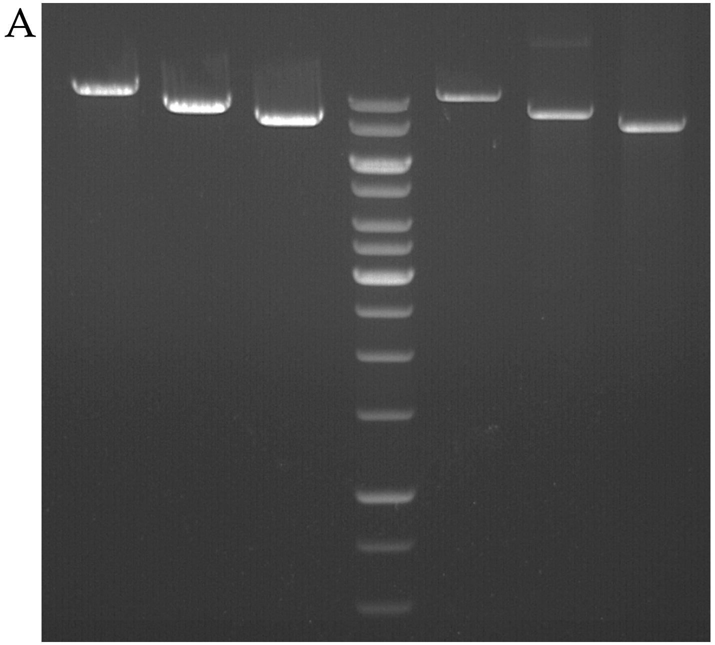

Digestion and gel electrophoresis of pLC70-derived plasmids.
(A) Plasmids digested with *Rru*I, recognising a single site on each plasmid.
Lane 1: pLC70 isolated from *E. coli*
Lane 2: pTNTrpE isolated from *E. coli*
Lane 3: pTNAg isolated from *E. coli*
Lane 4: GeneRuler 1kb DNA Ladder
Lane 5: pLC70 isolated from *T. kodakarensis*
Lane 6: pTNTrpE isolated from *T. kodakarensis*
Lane 5: pTNAg isolated from *T. kodakarensis*

B

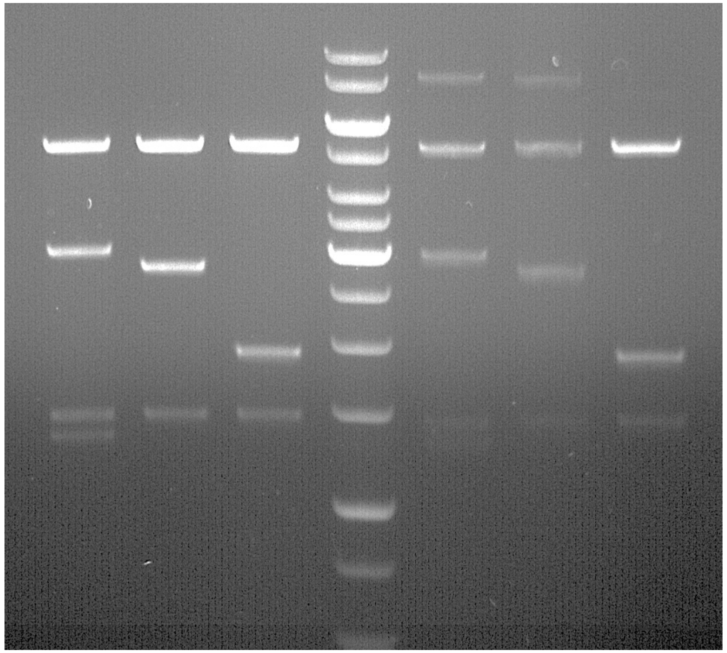

Digestion and gel electrophoresis of pLC70-derived plasmids.
(B) Plasmids digested with *Pst*I, recognising multiple sites on each plasmid.
Lane 1: pLC70 isolated from *E. coli*
Lane 2: pTNTrpE isolated from *E. coli*
Lane 3: pTNAg isolated from *E. coli*
Lane 4: GeneRuler 1kb DNA Ladder
Lane 5: pLC70 isolated from *T. kodakarensis*
Lane 6: pTNTrpE isolated from *T. kodakarensis*
Lane 5: pTNAg isolated from *T. kodakarensis*
Note that paritally digested plasmid forms an additional, high molecular weight band in T. kodakarensis preparations.
