# Supplementary material for: A series of new E. coli–Thermococcus shuttle vectors compatible with previously existing vectors
Source: Extremophiles. 2018 Mar 1;22(4):591–8. doi: 10.1007/s00792-018-1019-6 (PMC5988781; doi:10.1007/s00792-018-1019-6)
Supplement: Supplementary file 4 — Supplementary material 4 (PDF 37 kb) [file 792_2018_1019_MOESM4_ESM.pdf]

```
NC_021208    841 GCGTCCGGAAAAAGCGCGTGCAATACAGCTTCTTCGGTGTATAGATAACCCGTGAGGAAG  900
                 ||    |        || |  | |    |   |     |   |              |||
NC_021208    638 GCCAGAGTGGTCTCAGCATCAACTGGCTCCGGTAGCAAGCGGAAGACTGAGTATGCCAAG  697

NC_021208    901 CTTCGTCGGGATATCAACAAACTGAGGGATATGGGTCTTGTTTCGGTTGAAAAACTCGAT  960
                 ||||||||||||||||||||||||||||||||||||||||||||||||||||||||||||
NC_021208    698 CTTCGTCGGGATATCAACAAACTGAGGGATATGGGTCTTGTTTCGGTTGAAAAACTCGAT  757

NC_021208    961 GGCTTCCTCTGGGTGTCTCCTACTCCCAAGGCTCTTTACTTGATTGAACATAGGAGAAAT 1020
                 |||||||||||||||||||||||||||| |||||||||||||||||||||||||||||||
NC_021208    758 GGCTTCCTCTGGGTGTCTCCTACTCCCATGGCTCTTTACTTGATTGAACATAGGAGAAAT  817

NC_021208   1021 TCAAATCGTGTCCGTAGGTCGATGCGTCCGGAAAAAGCGCGTGCAATACAGCTTCTTCGG 1080
                 ||||||||||||||||||||||||||||||||||||||||||||||||||||||||||||
NC_021208    818 TCAAATCGTGTCCGTAGGTCGATGCGTCCGGAAAAAGCGCGTGCAATACAGCTTCTTCGG  877

NC_021208   1081 TGTATAGATATCCCGGGCTATTTTGTTTGGTCGAAAGATGGCAAGAACAAGGTTGCTGAG 1140
                 |||||||||| |||| |        |      || |      ||      | |    |
NC_021208    878 TGTATAGATAACCCGTGAGGAAGCTTCGTCGGGATATCAACAAACTGAGGGATATGGGTC  937
```

Supplementary Figure 1: Alignment of pTP2 sequence (NC_021208) with itself, showing the presence of a tandem repeat from nucleotide 898-1090 (695-887).
